# Supplementary material for: ALC1/eIF4A1-mediated regulation of CtIP mRNA stability controls DNA end resection
Source: PLoS Genet. 2020 May 11;16(5):e1008787. doi: 10.1371/journal.pgen.1008787 (PMC7241833; doi:10.1371/journal.pgen.1008787)
Supplement: S1 Table — (DOCX) [file pgen.1008787.s001.docx]

**Supplementary Table 1. siRNAs used in this study.**

| Target gene | Description/sequence (5’-3’) | Reference |
| --- | --- | --- |
| Non-Target | ON-TARGETplus Non-targeting Pool | D-001810-10-20 (Dharmacon) |
| CtIP | GCUAAAACAGGAACGAAUCTT | Custom siRNA (Sigma) |
| ALC1 | GGACAUGCCACGAAAGGUU | J-014368-07 (Dharmacon) |
| ALC1-2 | GGGAAGACCUGCCAGACUA | J-014368-05 (Dharmacon) |
| ALC1 3’UTR | GAGGUACUGCAAUAGAGUATT | SI05075805 (Qiagen) |
| eIF4A1 | CUGGCCGUGUGUUUGAUAUTT | Custom siRNA (Sigma) |
