# Supplementary material for: ALC1/eIF4A1-mediated regulation of CtIP mRNA stability controls DNA end resection
Source: PLoS Genet. 2020 May 11;16(5):e1008787. doi: 10.1371/journal.pgen.1008787 (PMC7241833; doi:10.1371/journal.pgen.1008787)
Supplement: S2 Table — WB: Western blot; IF; Immunofluorescence; SMART: Single Molecule Analysis of Resection Tracks. (DOCX) [file pgen.1008787.s002.docx]

**Supplementary Table 2. Primary antibodies used in this study.**

| Antibody | Species | Suppliers (Reference) | Application (dilution) |
| --- | --- | --- | --- |
| α-tubulin | Mouse | Sigma (T9026) | WB (1:50,000) |
| β-Actin | Rabbit | Abcam (Ab8227) | WB (1:20,000) |
| HSP70 | Mouse | Santa Cruz (sc-24) | WB (1:20,000) |
| ALC1 | Mouse | Santa Cruz (sc-81065) | WB (1:250) |
| BrdU | Mouse | Amersham (RPN202) | SMART (1:500) |
| γH2AX | Rabbit | Cell Signaling (2577L) | IF (1:500) |
| γH2AX | Mouse | Millipore (05-525) | IF (1:50 |
| RPA32 | Mouse | Abcam (ab2175) | IF (1:500) |
| CtIP | Mouse | Kind gift from R. Baer | WB (1:500) |
| 53BP1 | Rabbit | Novus (NB100-304) | IF (1:500), WB (1:1,000) |
| RIF1 | Goat | Santa Cruz (sc-55979) | IF (1:100) |
| CCAR2 | Rabbit | Bethyl (A300-433A-1) | IF (1:150), WB (1:1,500) |
| eIF4A1 | Rabbit | Abcam (ab31217) | WB (1:1,000) |
| CENPF | Rabbit | Abcam (ab5) | IF (1:500) |
| GFP | Rabbit | Santa Cruz (sc-8334) | WB (1:1,000) |
