# Supplementary material for: ALC1/eIF4A1-mediated regulation of CtIP mRNA stability controls DNA end resection
Source: PLoS Genet. 2020 May 11;16(5):e1008787. doi: 10.1371/journal.pgen.1008787 (PMC7241833; doi:10.1371/journal.pgen.1008787)
Supplement: S3 Table — WB: Western blot; IF; Immunofluorescence; SMART: Single Molecule Analysis of Resection Tracks. (DOCX) [file pgen.1008787.s003.docx]

**Supplementary Table 3. Secondary antibodies used in this study.**

| Antibody | Species | Suppliers (Reference) | Application (dilution) |
| --- | --- | --- | --- |
| Alexa Fluor 594 anti-mouse | Goat | Invitrogen (A11005) | IF, SMART (1:1,000) |
| Alexa Fluor 488 anti-rabbit | Goat | Invitrogen (A11034) | IF (1:1,000) |
| Alexa Fluor 647 anti-rabbit | Goat | Invitrogen (A21244) | IF (1:1,000) |
| Alexa Fluor 568 anti-mouse | Goat | Invitrogen (A11044) | IF (1:1,000) |
| Alexa Fluor 594 anti-goat | Donkey | Invitrogen (A11058) | IF (1:1,000) |
| Alexa Fluor 488 anti-rabbit | Donkey | Invitrogen (A21206) | IF (1:1,000) |
| IRDye 680RD anti-mouse IgG (H+L) | Goat | LI-COR (926-68070) | WB (1:5,000 – 1:10,000) |
| IRDye 800CW anti-rabbit IgG (H+L) | Goat | LI-COR (926-32211) | WB (1:5,000 – 1:10,000) |
