# Supplementary material for: ALC1/eIF4A1-mediated regulation of CtIP mRNA stability controls DNA end resection
Source: PLoS Genet. 2020 May 11;16(5):e1008787. doi: 10.1371/journal.pgen.1008787 (PMC7241833; doi:10.1371/journal.pgen.1008787)
Supplement: S4 Table — (DOCX) [file pgen.1008787.s004.docx]

**Supplementary Table 4. Primers used in this study.**

| Primer name | Sequence (5’-3’) | Use |
| --- | --- | --- |
| ACTB qPCR Fw | ACGAGGCCCAGAGCAAGA | RT-qPCR of β-Actin as housekeeping |
| ACTB qPCR Rv | GACGATGCCGTGCTCGAT | RT-qPCR of β-Actin as housekeeping |
| ALC1 qPCR Fw | CTGCCGATGCTGACCTCCAG | RT-qPCR of ALC1 to check depletion |
| ALC1 qPCR Rv | TCCTTCCTCTTGATCTCTGCTCC | RT-qPCR of ALC1 to check depletion |
| RT-CtIP Rv | CTAGTCTTTGTGCATCTAAG | RT of CtIP mRNA specifically |
| BglII G4less Fw | GGTGGTAGATCTGCGCCGACTGCGGCTC | Amplification of G4less CtIP 5’UTR adding restriction site for BglIII enzyme (cloning) |
| XhoI G4 Fw | GGTGGTCTCGAGTGGAACTCCCGCGTGAC | Amplification of G4 CtIP 5’UTR adding restriction site for XhoI enzyme (cloning) |
| HindIII common Rv | CCACCAAAGCTTGCTTAATATGCTCCACACTTCTACTTGC | Amplification of G4less/G4 CtIP 5’UTR adding restriction site for HindIII enzyme (cloning) |
| pGFP-Cter Fw | CATGGTCCTGCTGGAGTTCGTG | qPCR of pGFP |
| pGFP-Cter Rv | CTCGTCCATGCCGAGAGTGATC | qPCR of pGFP |
| rRNA18S Fw | CTCAACACGGGAAACCTCAC | RT-qPCR of rRNA18S as housekeeping |
| rRNA18S Rv | CGCTCCACCAACTAAGAACG | RT-qPCR of rRNA18S as housekeeping |
| ACTB-PCR Fw | GATGACCCAGATCATGTTTGAG | PCR of β-Actin as positive control for cloning |
| ACTB-PCR Rv | GACTCCATGCCCAGGAAG | PCR of β-Actin as positive control for cloning |
| 5’UTR GFP Seq | GCAGAGCTGGTTTAGTGAAC | DNA sequencing of GFP constructs |
| G4less Fw | GATGGAGAAGGGACCTGGCTG | qPCR of G4less CtIP mRNA to check expression level |
| G4 Fw | GGAGGGGTCGGCTTTCCCAC | qPCR of G4 CtIP to check mRNA expression level |
| G4less/G4 Rv | GTCGTCTTTGGACAGGTCAAATACC | qPCR of G4less/G4 CtIP to check mRNA expression level |
| CtIP qPCR Fw | AGAAATTGGCTTCCTGCTCAAG | RT-qPCR of CtIP to check depletion |
| CtIP qPCR Rv | GAAAACCAACTTCCCAAAAATTCTC | RT-qPCR of CtIP to check depletion |
