# Supplementary figures and images for: ALC1/eIF4A1-mediated regulation of CtIP mRNA stability controls DNA end resection
Source: PLoS Genet. 2020 May 11;16(5):e1008787. doi: 10.1371/journal.pgen.1008787 (PMC7241833; doi:10.1371/journal.pgen.1008787)

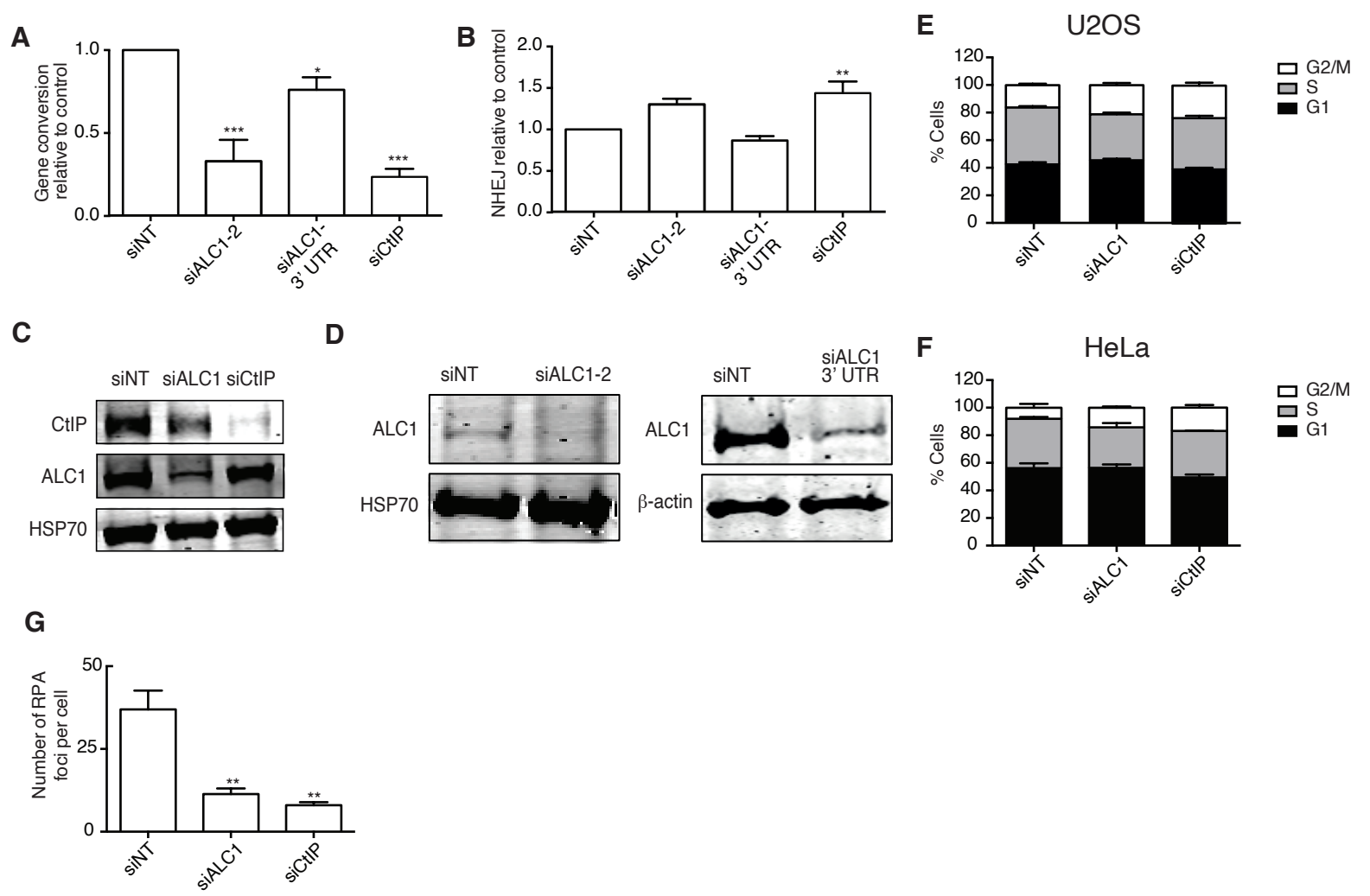

Supplement: S1 Fig — A, Gene conversion, measured as described in Fig 1A in cells depleted of ALC1 using two additional siRNAs. siALC1-2 targets the coding region, whereas siALC1- 3’ UTR targets the 3’ UTR of the RNA. B, Same as A, but using the EJ5 reproter to check NHEJ efficiency. C, Depletion efficiency of ALC1 in cells transfected with the indictaed siRNAs. D, Same as A, but using additional siRNA against ALC1, either ALC1-2 (left) or an siRNA targeting the 3’ UTR (right) in U2OS cells. E, Cell cycle profile of U2OS cells transfected either with siNT control sequence or with siRNAs against ALC1 and CtIP as indicated. The average and standard deviation of three independent experiment are plotted. F, Same as C but in HeLa cells. G, The average number of RPA foci per cell formed 1h upon exposure to 10Gy of ionizing radiation was scored automatically using the software Metamorph. The average and standard deviation of three independent experiments are plotted (left) and representative images of one cell are shown on the right. Statistical analysis as described in Fig 2A. (PDF) [file pgen.1008787.s005.pdf]

**A**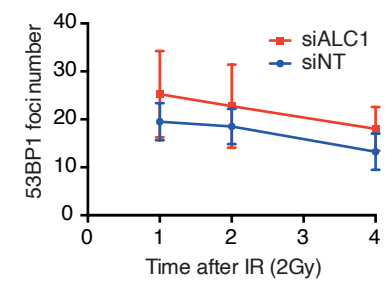**B**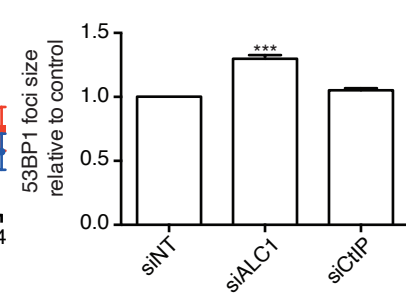**C**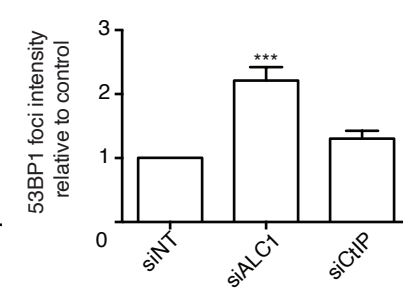**D**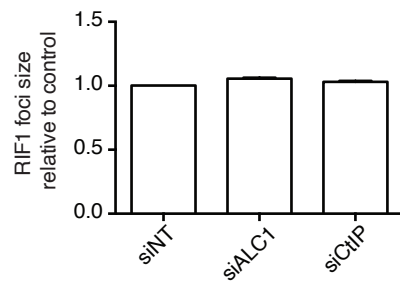**E**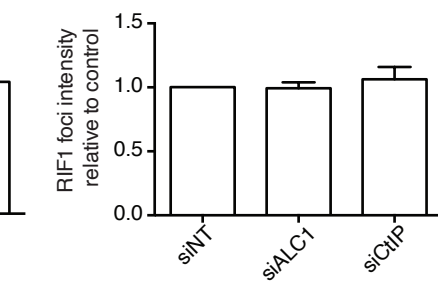**F**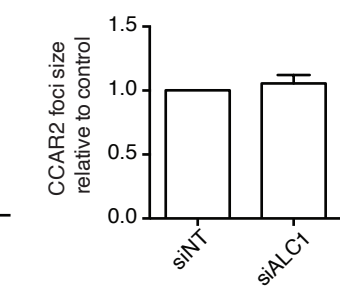**G**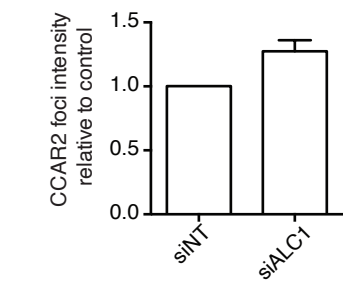

Supplement: S2 Fig — A, The average number of 53BP1 foci per cell was calculated as described in Fig 3A in cells at different times after exposure to 2 Gy of ionizing radiation in control cells and cells depleted for ALC1 (red). B, Computer-based measurement of the size of 53BP1 foci in cells depleted for the indicated factors using the Metamorph software. Size was normalized with the control sample. The average and standard deviation of three independent experiments is plotted. Other details as Fig 1A. C, Computer-based analysis of the intensity of 53BP1 foci. Other details as panel B. D, Same as B but for RIF1 foci. E, Same as C but for RIF1. F, Same as B but for CCAR2 foci. G, Same as C but for CCAR2. (PDF) [file pgen.1008787.s006.pdf]

**A**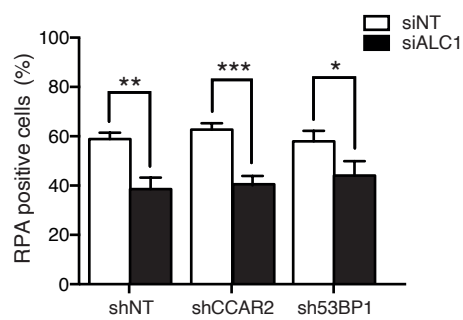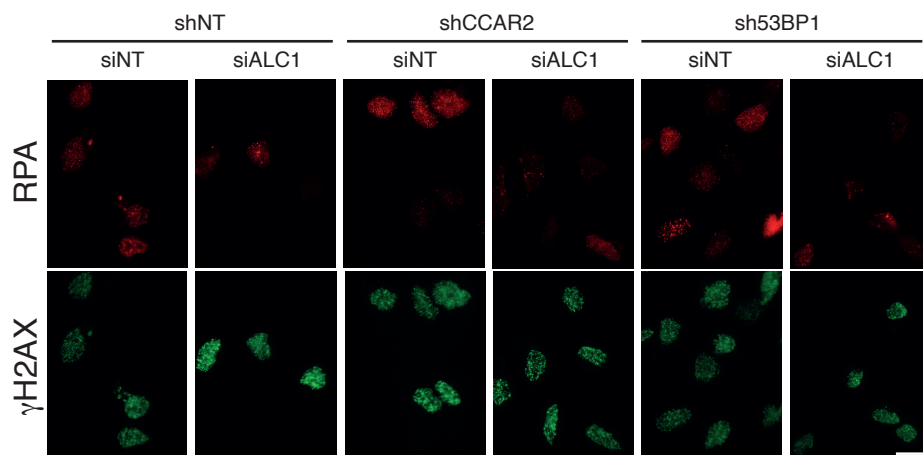**B**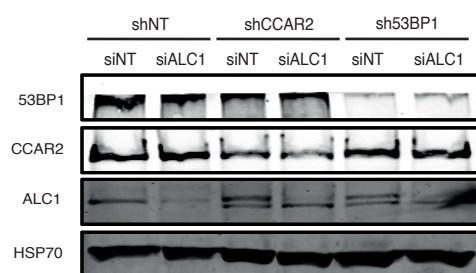**C**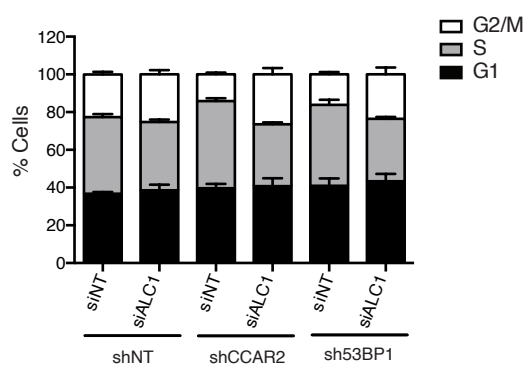

Supplement: S3 Fig — A, Resection was measured 1h after irradiation in cells infected with shRNA against 53BP1, CCAR2 or a control sequence and transfected with an siRNA against either ALC1 (black bars) or a control sequence (white bars). Representative images are shown on the right. Scale Bar represent 25 μm. Other details as in Fig 5B. B, Western blot showing the downregulation of ALC1, CCAR2 and 53BP1 of the cells described in A. HSP70 was used as loading control. C, Cell cycle analysis of the cells described in panel A. (PDF) [file pgen.1008787.s007.pdf]

**A**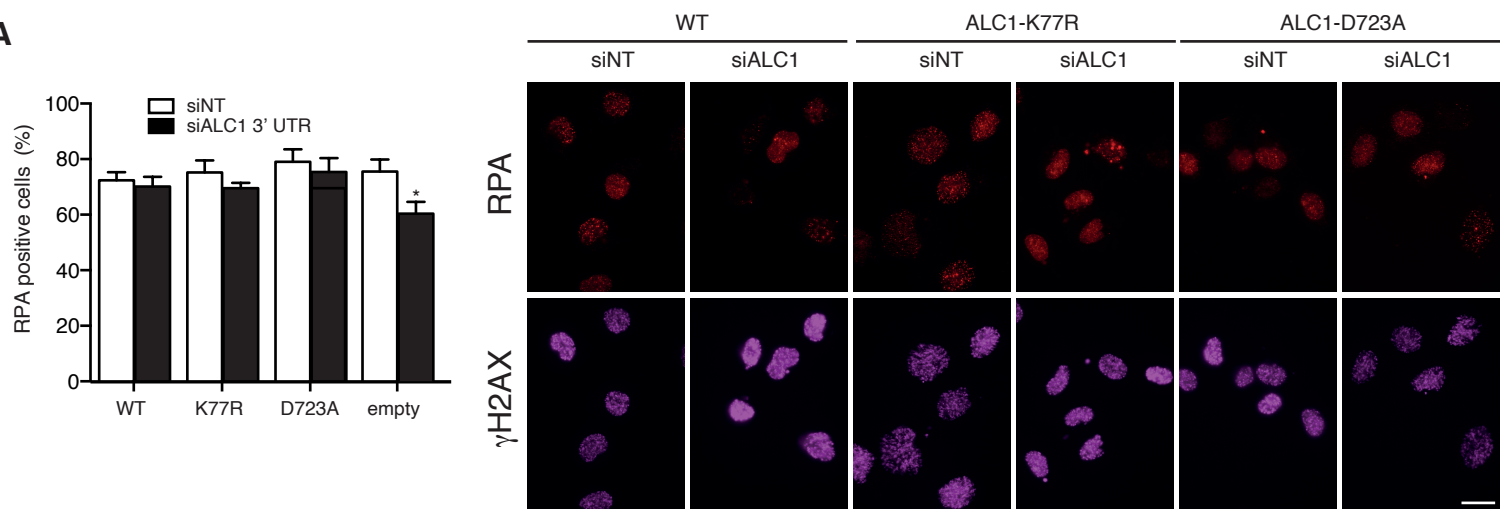**B**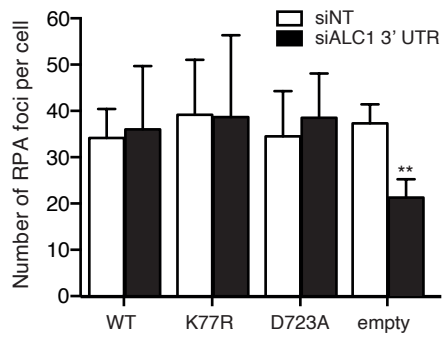**C**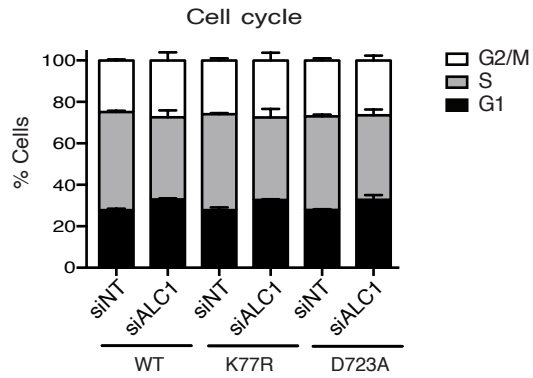**D**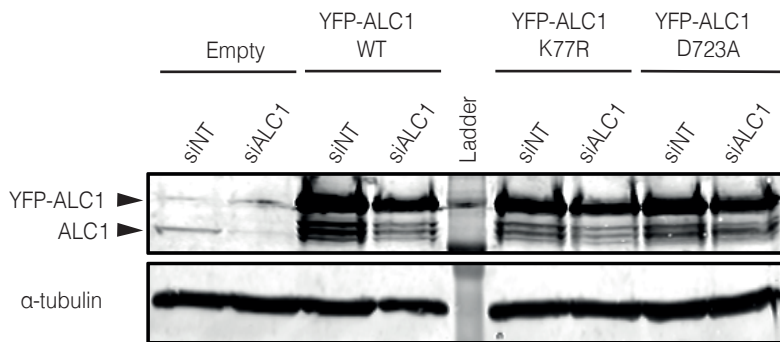**E**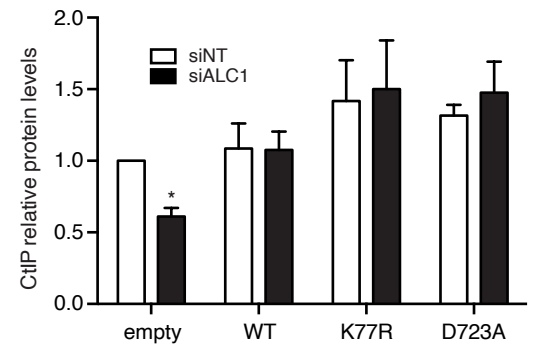

Supplement: S4 Fig — A, Resection was measured 1h after irradiation in cells transfected with siRNA against ALC1 (black bars) or a control sequence (white bars) and then transfected with the different YFP-ALC1 constructs as mentioned in Fig 2. Representative images are shown on the right. Scale Bar represent 25 μm. Other details as in Fig 2A. B, The average number of RPA foci per cell in samples treated as in panel A and formed 1h upon exposure to 10Gy of ionizing radiation was scored automatically using the software Metamorph. The average and standard deviation of three independent experiments are plotted. C, Cell cycle analysis of the cells described in panel A. D, Western blot showing the expression of YFP-tagged version of ALC1. Protein samples from cells transfected with the indicated ALC1 variants, downregulated or not for the endogenous version using siRNA as depicted in the figures, were resolved in SDS-PAGE and blotted with an ALC1 antibody. The YFP-tagged and endogenous proteins are marked in the blot with triangles. α-tubulin was used as loading control. E, The levels of CtIP protein in cells transfected with the mentioned version of YFP-ALC1 and depleted (black bars) or not (white bars) of endogenous ALC1 with an siRNA targeting the 3’ end of the mRNA was determined by Western blot quantification using the Odissey Li-Cor Infrared system and normalized to control cells transfected with an empty plasmid. The average and standard deviation of three independent experiment are plotted. (PDF) [file pgen.1008787.s008.pdf]

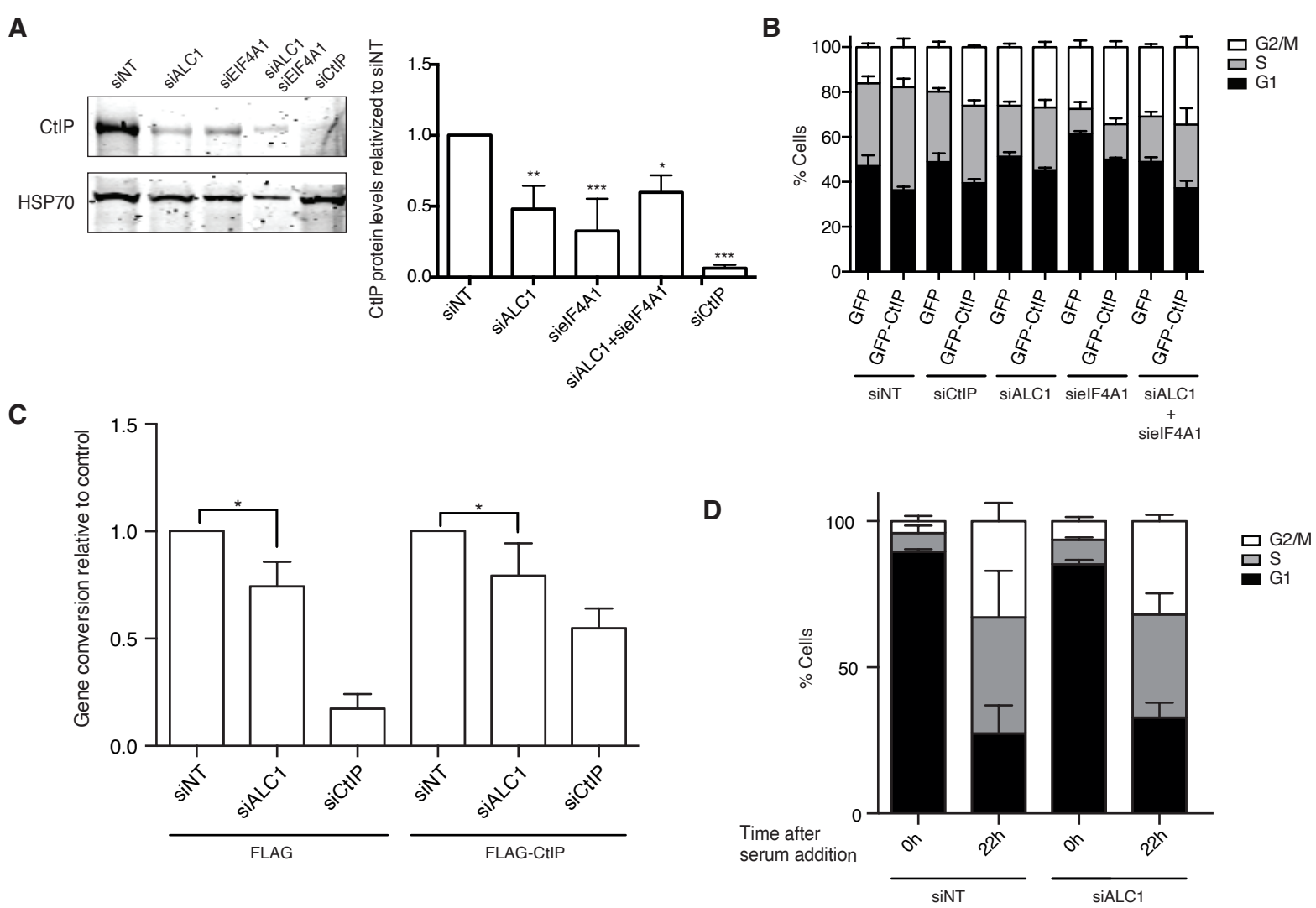

Supplement: S5 Fig — A, The levels of CtIP protein in cells depleted for the indicated proteins was determined by western blot quantification using the Odissey Li-Cor Infrared system and normalized to control cells. A representative image is shown on the right side and the average and standard deviation of three independent experiment are plotted on the left. Statistical significance was calculated using a one-way ANOVA test. One, two or three asterisks represent p<0.05, p<0.01 and p<0.001, respectively. B, Cell cycle analysis of the cells described in Fig 5B. C, Gene conversion was measured as described in Fig 1A in cells bearing with a FLAG-CtIP or an empty FLAG plasmid and transfected with the indicated siRNAs, D, RPE1 cells synchronized by serum starvation and release as described in the methods section were collected 0h or 22h after serum addition and cell cycle distribution was assayed by FACs analysis. At 0h an enrichment in G1 cells was observed, whereas 22 h later an enrichment on S and G2 was detected. (PDF) [file pgen.1008787.s009.pdf]
