# Supplementary material for: ALC1/eIF4A1-mediated regulation of CtIP mRNA stability controls DNA end resection
Source: PLoS Genet. 2020 May 11;16(5):e1008787. doi: 10.1371/journal.pgen.1008787 (PMC7241833; doi:10.1371/journal.pgen.1008787)
Supplement: Attachment — Submitted filename: rebuttal.docx [file pgen.1008787.s010.docx]

We would like to tank both reviewers for giving us the opportunity to address their concerns in a revised version. A point-by-point response to the issues raised by both referees follows.

*Reviewer #1: The manuscript by Mejias-Navarro identifies a novel mechanism that controls DNA end resection by regulating the expression of a key resection factor CtIP. It is demonstrated that ALC1 has a structural role, together with the transcription factor eIF4A1, to regulate the stability of the major mRNA coding for CtIP. Specifically, the depletion of either ALC1 or eIF4A1 decreases the stability of CtIP mRNA. Interestingly, the effect is linked to a 5' UTR of mRNA CtIP, and, elegantly, it is demonstrated that fusing this UTR (containing a G-rich sequence) to an unrelated mRNA results in the same regulatory effect.*

We would like to thank this reviewer for his/her positive revision of the manuscript. We have answered his/her concern in detail below.

*The works is generally well-done. One conceptual point that is missing is a lack of understanding when and where such a regulation would take place. Dr. Huertas earlier showed that Sae2 and CtIP are regulated by post-translational manner by phosphorylation, which links cell cycle regulation to resection, and which generally allows resection when sister chromatids are available. Furthermore, resection is further e.g. regulated by ATM phosphorylation of CtIP, which responds to the presence of DNA damage. Can the authors comment on what function the mechanism involving ALC1/eIF4A1 has? Is it cell cycle or DNA damage regulated?*

This is a very interesting point, unfortunately we have not been able to find the conditions in which each isoform of CtIP is specifically regulated. The same effect of ALC1 depletion can be observed on each CtIP isoform in G1 arrested cells or samples enriched for S and G2 cells (new Figure 6D and E, supplementary Figure 5D). Regarding the response to DNA damage, that is something we are currently exploring in more detail, but we do not see a short-term response in that regard.

*Furthermore, while I am in most cases convinced by the data presented, one exception is the quantitation of RPA positive cells. Looking at the representative images shown, it seems to be very difficult to decide whether a cell is "positive" or "negative", as there appears to be a whole range of RPA intensities from almost invisible to very strong. It would be more convincing to show FACS profiles for some of the key experiments (Fig. 1 and Fig. 4).*

We understand the referee concern. However, we have never been able to test resection using RAP in FACs in the lab, probably due to technical issues outside of our control. I want to point out that the resection phenotype is not only based in RPA positive cell scoring in different cell lines, although our lab is an expert in that technique, but it is also supported by SMART, that is a higher resolution technique than both RPA foci and FACs and by un unbiased counting of the average number of RPA foci per cell (Supplementary figure 4B). Thus, we think that this point is sufficiently clear as it is.

*The authors conclude that the "5' UTR of CtIP is sufficient to render the stability of any mRNA", while in fact only one other RNA was tested. It would certainly be interesting to perform transcriptome-wide analysis upon ALC1/eIF4A1 depletion and correlation with the likelihood of G4 being formed at UTR; otherwise, the authors should rephrase the statement.*

It is true that it will be interesting to do a genomewide analysis of the effect of ALC1, but we think it is under the scope of the current manuscript. Therefore, and as suggested by the reviewer, we have toned down our claim in the revised version.

*Below are minor points and typos I found while the reading the text

page 3: "this include the activation"
page 9: Fig 1A and B, panels A and B
page 10: Fig Supplementary Fig
page 10: We observed a reduced not only in the number
page 12: Fig. 5A does not show double-depletion
page 13: CtIP gene produce two different*

Thank you very much for pointing out those typos. We have corrected them in the revised version.

*Reviewer #2: The study from Mejías-Navarro and colleagues report that loss of ALC1 (also called CHDL1) reduces homologous recombination (HR), which they attribute to reduced end-resection measured by RPA foci upon DNA damage. In this context, they find that ALC1 knockdown reduces the protein level of CtIP, a factor implicated in the initiation of DNA end-resection. Surprisingly, their data suggest that ALC1 regulates CtIP level independently from its enzymatic activity or PAR-binding. They confirm the interaction between ALC1 and eIF4A1 that previously identified in proteomic screens. It is known that eIF4A1 is an RNA helicase implicated in the resolution of G4 RNA structure. Using bioinformatic tools, they identify 2 isoforms of CtIP mRNA with different 5’UTR sequences due to alternative splicing. They suggest that ALC1 and eIF4A1 regulate the stability of the CtIP isoform with the G4 structure. Analyses of the GFP with G4 or G4less 5’UTR derived from CtIP confirm the findings. Overall, the study identified a new mean to regulate end-resection, by modulating the stability of CtIP mRNA through ALC1 and eIF4A1. The strengths are the novel mechanism and the potential chromatin independent role of ALC1 in DNA repair. CtIP protein levels are low in G1 and much higher in S/G2. It is important to clarify whether the different isoforms of CtIP transcripts present in different cell cycle phases and whether ALC1 and eIF4A1 have cell cycle independent roles on CtIP level regulation. Meanwhile, whether the G4-mediated regulation of CtIP contributes to the cell cycle phase dependent regulation of CtIP protein level. In addition, there are a few other concerns limited the enthusiasm to the manuscript.*

Thank you very much for the balanced review of our manuscript. We have aimed to solve all the issues raised.

*Major concerns:
1) The observations on the un-coordinate changes between CtIP mRNA level and protein level upon ALC1 or eIF4A1 depletion are quite interesting. This is also true for cell cycle specific regulation of CtIP protein levels. Sup Figure 5B showed that eIF4A1 knockdown caused significant G1 and G2/M arrest in the cells test. It is important to understand whether eIF4A1 can directly regulate CtIP stability independent of the cell cycle phases.*

This is an important point raised by the referee. We have performed cell cycle synchronization of cells depleted for ALC1 or eIF4A1 in U2OS and RPE cells. In U2OS cells we tried thymidine-nocodazol synchronization and in RPE1 cells we synchronized by serum starvation and then release by serum addition. Regarding eIF4A1, we had problems releasing the cells from any of those cell cycle synchronization protocols and an important fraction of the cells downregulated for eIF4A1 stayed in G1 upon synchronization. Therefore, this preclude us to address directly that effect. However, for cells depleted for ALC1 we were able to obtain a similar enrichment of specific cell cycle stages than in cells transfected with a control siRNA. The results were more consistent with RPE1 cells upon serum starvation, so we included those data in the manuscript, but similar results, albeit with a higher variability, were obtained in U2OS cells upon thymidine-nocodazole block. As shown in the new figure 6D and 6E and in supplementary fig 5D, ALC1 affected specifically the G4-containing transcript in a similar fashion, regardless of the cell cycle stage.

*2) Can CtIP expression rescue the HR defects in ALC1 deficient cells? This experiment is important to establish CtIP reduction as the main mechanism by which ALC1 contributes to HR.*

We have performed the experiments as required and, interestingly, FLAG-CtIP cannot rescue the HR defect, pointing out that indeed there are additional roles of ALC1 in homologous recombination. This probably reflects the chromating-remodelling role of ALC1 or its regulation of additional HR factors. Those data have been included and discussed in the revised version.

*3) In figure 2D, the impact of ALC1 knockdown has a much more dramatic effect on RPA foci formation in telomerase positive Hela cells, than ALT- U2OS cells. Does the two form of CtIP transcripts also exist in Hela cells and are they also protected from ALC1 and eIF4A?*

Both isoform indeed exist in HeLa cell lines (and in RPE1 as mentioned above), and they are similarly affected by ALC1 depletion (See revised version, new figure 6B-E).

4) Figure 5C showed that ectopic expression of CtIP without the 5’UTR rescued the resection measured in siALC1 cells. How about the CtIP cDNA with endogenous 5’-UTR?

We tried this experiment by creating different constructs with the different UTRs. Indeed, it has taken us months to obtain them. Once we obtained plasmids that were expressing the desired *CtIP* mRNAs we tested that the GFP-CtIP protein was expressed and that the responded as expected to ALC1 and eIF4A1 depletion:

Unfortunately, and for unknown reasons to us, those constructs seem non-functional, as they cannot rescue either the depletion of CtIP or ALC1

Thus, unfortunately, we could not obtain meaningful results with those constructs. As we have tried many different cloning strategies, we think that adding those UTRs in our cDNA CtIP is rendering us not functional fusions for some unknown reason.

*Minor comments:
1) Page 4 – line 4 “…… the presence of DSBs triggers a complete, massive cellular response.” Should it be complex, instead of complete?*

Yes, thank you for pointing out.

*2) The author should tone down the discussion regarding the chromatin dependent function of ALC1. Although the CtIP mRNA regulation by ACL1 seems independent of it helicase activity or the Macro domain, there is no direct evidence in the manuscript to exclude the possibility that ALC1 might also promote HR through other mechanisms beyond regulating CtIP mRNA.*

The referee is actually right. We have changed the discussion to imply that the resection effect is due to CtIP regulation, but that the HR effect is indeed more complex, and that there are additional roles for ALC1 during this process, including most likely effects dependent on the chromatin remodeling role of ALC1.

*3) There is some inconsistency on whether CtIP G4-mRNA is more stable or at higher accumulative level. It would be helpful to clarify this when it was first measured.*

We measure mainly steady state levels, so in that regard is difficult to conclude if the mRNA is more stable or at a higher accumulative level. Considering than in siNT transfected cells ActD treatment has little effect in the G4less and some effect on the G4, I will argue that is indeed slightly less stable but accumulates at a higher level. We have tried to clarify this in the text.

*4) The official gene number of ALC1 is CHDL1. It would be preferred to use CHDL1.*

I am happy to use either, depending on what the editor suggests. We used ALC1 as it was known as such in the DNA Damage response field, but we can change it throughout the manuscript if the editor says so.
